# *Pseudomonas fluorescens* CRBSI outbreak: complying with the standardization of invasive procedures is a step ahead in the fight against antimicrobial resistance

**DOI:** 10.1186/s13756-024-01390-9

**Published:** 2024-04-12

**Authors:** Patricia Volkow, Tania Guadalupe Chávez-Chávez, Bertha García-Pineda, Consuelo Velázquez-Acosta, Daniel Carpio-Guadarrama, Diana Vilar-Compte, Cyntia Ibanes-Gutiérrez

**Affiliations:** 1 Department of Infectious Diseases, Instituto Nacional de Cancerología, Av. San Fernando No. 22, Col. Belisario Domínguez, Sección XVI, Tlalpan, Mexico City, 14080 Mexico; 2 Infection Prevention and Control Team, Instituto Nacional de Cancerología, Av. San Fernando No. 22, Col. Belisario Domínguez, Sección XVI, Tlalpan, Mexico City, 14080 Mexico; 3 Microbiology Laboratory, Instituto Nacional de Cancerología, Av. San Fernando No. 22, Col. Belisario Domínguez, Sección XVI, Tlalpan, Mexico City, 14080 Mexico

**Keywords:** Antimicrobial resistance, Catheter-related bloodstream infection, CRBSI, Hospital acquired infection, *Pseudomonas fluorescens*

## Abstract

In the healthcare sector, the implementation of standardized procedures, such as those commonly employed in franchises to ensure consistent quality, remains underprioritized. Within this framework, we focus on the importance of standardized central venous catheter (CVC) insertion procedures to prevent healthcare-associated outbreaks. While antimicrobial resistance (AMR) may still not be the most prevalent problem in some institutions, its increasing significance certainly underlines the urgency of infection prevention.

We aim to highlight this issue by describing and discussing an outbreak scenario of carbapenem-resistant (CR) *Pseudomonas fluorescens* bloodstream infections resulting from a deviation from the standardized CVC insertion procedure. This outbreak led to six episodes of catheter related bloodstream infection (CRBSI) in patients with hematologic malignancies, delaying their primary treatment. Nineteen patients were exposed, leading to an attack rate of 31.6%.

## Background

A franchise is a license that allows someone to use the expertise and methods of an owner to guarantee that a service or a product is delivered with consistent quality. This concept of standardization has yet to spread further into healthcare delivery as a preventive strategy for healthcare-associated infections, which is, in turn, crucial in the fight against antimicrobial resistance (AMR).

Although a central venous catheter (CVC) is to be installed using a sterile technique, the technical personnel, supplies, and procedures vary between hospitals and countries. The World Health Organization has dictated that poor infection prevention and control in healthcare facilities contributes to the emergence of AMR [1]. If a procedure, like CVC insertion, is properly done, a patient will have a much lower risk of getting an infection that will need antibiotics and thus will have a lesser exposure to antimicrobial selective pressure.

This study describes how the breach of a long-time standardized procedure resulted in a catheter-related bloodstream infection (CRBSI) outbreak by carbapenem-resistant (CR) *Pseudomonas fluorescens*.

## Material and methods

### Setting and population

An increase in the number of *P. fluorescens* isolates in blood cultures was noted in June 2022, leading to a retrospective clinical chart review of patients with a positive culture. Demographic and clinical data and data regarding microbiologic culture results were retrieved for each patient. This information included name, diagnosis, chemotherapy regimen, invasive procedures, date of positive blood culture, isolated microorganism, and antibiogram; it was recorded in a spreadsheet. The common characteristic among patients with positive cultures was a recent history of external double-lumen CVC installation.

The Instituto Nacional de Cancerología (INCan) in Mexico is a 133-bed referral hospital for oncologic adult patients. A team of nine nurses exclusively dedicated to standardized intravenous (IV) therapy, including assistance in CVC insertion, has been in place since 1990. Senior IV team nurses within our institution provide the training for these nurses. Additionally, our suppliers conduct specific training for the insertion of PICC lines. It's important to note that nurses must complete this training before they can join the IV-team. Regarding the location of CVC installations, while some are performed in the IV-team unit, others, particularly those for patients with difficult vascular access, are conducted in the Interventional Radiology Unit (IRU). This decision is based on clinical judgment and each patient's specific needs. In the IRU, the institutional standardized procedure for CVC placement involves insertion by an interventional radiologist and assistance throughout the installation process by a trained IV-team nurse to ensure adherence to sterile procedures [2–4]. Since 2020, the annual average number of CVCs installed has been 1869 (282 one-lumen, 512 double-lumen, 31 triple-lumen, 79 high-flux, and 966 implanted ports). The rate of CRBSI during the year preceding the outbreak was 1.92 per 1000/catheter days; during the month of the outbreak, it was 9.62.

### Case definition

A confirmed case was defined as any patient with a positive blood culture for *Pseudomonas fluorescens* after undergoing CVC insertion between June 21 and July 20, 2022, in the IRU of the INCan in Mexico City, Mexico.

### Microbiology

Blood samples were cultured in BD BACTEC™ FX System (Becton Dickinson Microbiology Systems, USA), and plated on blood, and MacConkey agar. Isolated bacteria were identified using the Bruker Daltonics IVD MALDI Biotyper®. Identification scores of ≥ 2.0 were used to determine species-level identification, which were attained in every sample in which *P. fluorescens* was isolated. Antimicrobial susceptibility testing was determined in accordance with Clinical and Laboratory Standards Institute (CLSI) guidelines utilizing Vitek2™ semi-automated testing.

### Outbreak investigation

In July 2022, CR *P. fluorescens* were recovered from blood cultures from recently placed external double-lumen CVCs. The outbreak was confirmed by revision of the microbiologic culture archives, in which no *P. fluorescens* had been isolated from blood samples in the ten-year preceding period at our institution. The investigation was carried out by the hospital’s Infection Prevention and Control (IPC) team. It involved a comprehensive review of patient records and laboratory results and direct observation of the CVC placement procedure in the IRU, during which deviations in the standardized process were noted to request environmental cultures in an orderly manner.

## Results

Six episodes of CR *P. fluorescens* CRBSI were confirmed in five patients with hematological malignancies. One of the patients had his CVC removed due to the CRBSI diagnosis, and then another CVC was inserted after the 14-day time-frame period had elapsed. He subsequently developed another CRBSI episode that was included in the outbreak as an additional event since he met the case definition again after having negative blood cultures between each event. The patients’ characteristics are summarized in Table 1.

**Table 1:** Patient characteristics during the CR *P. fluorescens* CRBSI outbreak in chronological order of CVC insertion date

Patient	Number of CRBSI per patient	Age	Sex	Diagnosis	Date^a^ of CVC insertion	Date^a^ of blood culture withdrawal	Time to positivity of blood culture (hours)
*1*	1	78	Male	ALL/LBL	21/06/2022	15/07/2022	64
*2*	1	33	Female	AML	27/06/2022	16/07/2022	12
*3*	1	33	Female	AML	13/07/2022	15/07/2022	20
*4*	1	79	Male	DLBCL	15/07/2022	26/07/2022	24
*5*	1	74	Female	HL	19/07/2022	27/07/2022	24
*3*	2	33	Female	AML	27/07/2022	27/07/2022	12

*ALL/LBL* Acute Lymphoblastic Leukemia/Lymphoblastic Lymphoma, *AML* Acute Myeloid Leukemia, *DLBCL* Diffuse Large B-Cell Lymphoma, *HL* Hodgkin’s lymphoma

^a^Dates are presented in dd/mm/yyyy format

The investigation found that hand hygiene and scrubbing processes were correctly performed, as was skin antisepsis at the CVC insertion site. However, the IPC team uncovered breakdowns in the standardized CVC insertion procedure: IV-team nurses had stopped consistently assisting the CVC placement procedure a few weeks before the outbreak was identified, and instead, a non-IV therapy trained nurse assisted during installation. Sterile surgical drapes and sterile material were placed in an instrument table according to the standardized procedure except for an irrigation solution contained in an open, refilled, unlabelled bottle, which was poured into a sterile surgical bowl. This non-sterile solution was used to lubricate the CVC before placement by the non-IV therapy trained nurse. This action is not part of the standardized protocol and was thus cultured as it was suspected to be a possible source of the outbreak.

**Fig. 1:**
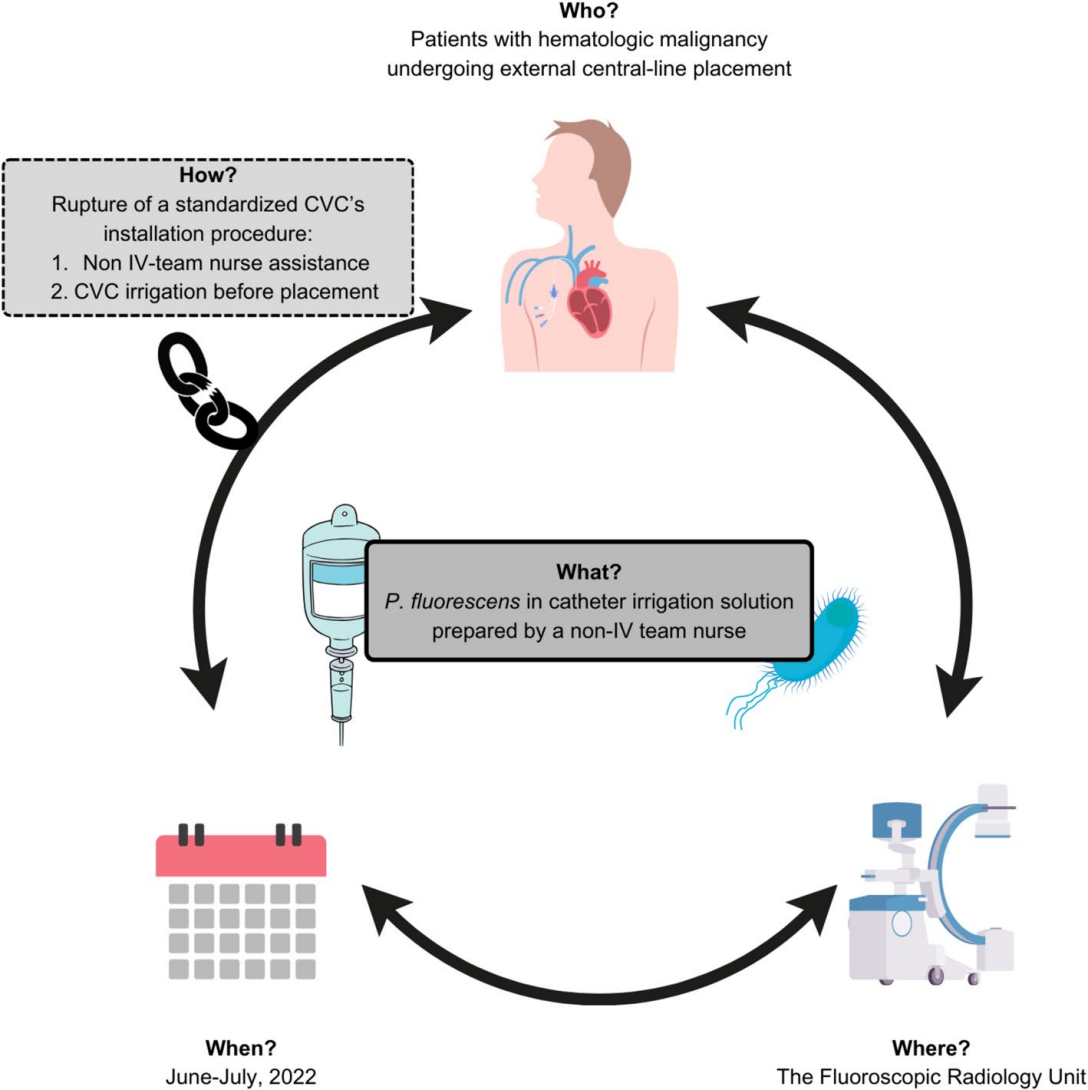
*P. fluorescens* CRBSI outbreak: breach of the CVC installation standardized procedure

The involvement of the non-IV therapy trained nurse that participated in the insertion of CVCs was a result of staffing shortages and excessive workload. The issue regarding the non-sterile solution used by interventional radiologists during CVC irrigation was a critical oversight. The non-trained nurse, lacking the necessary training, did not recognize this deviation from sterile protocol. CR-*P. fluorescens* was isolated from this solution and had the same antibiogram as all blood isolates. The outbreak is summarized in Fig. 1.

**Fig. 2:**
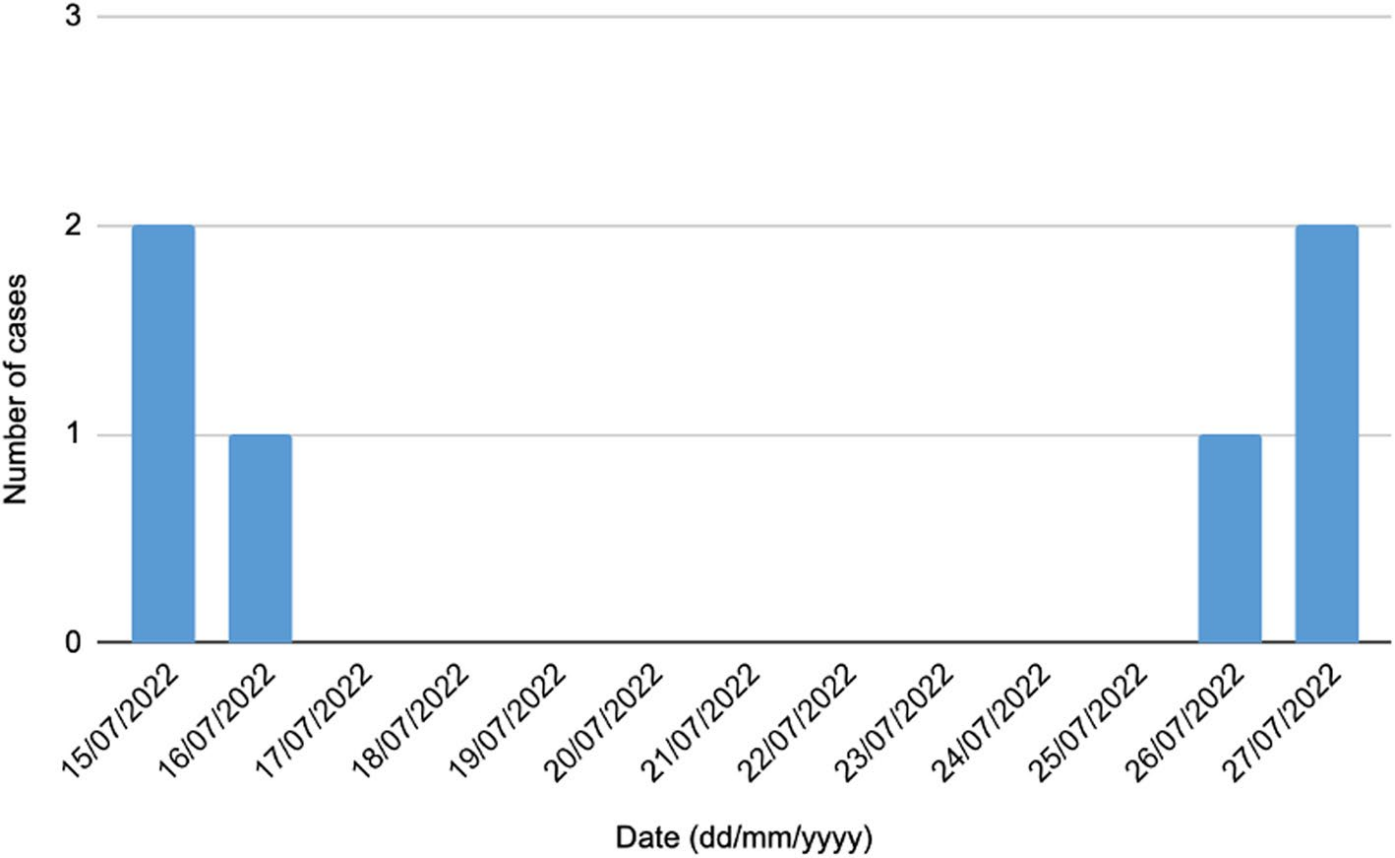
Epidemic curve of the CR *P. fluorescens* CRBSI outbreak

Once the source of the outbreak was identified, central and peripheral blood cultures were obtained from all patients who had undergone CVC placement in the IRU from June 21 and July 27, 2022. Forty CVCs were placed in the IRU during this period, 21 port catheters and 19 external-CVCs. No port catheter was infected; as, the IV-team nurse always assisted during port-catheter placement, while assistance by the non-IV therapy trained nurse occurred during placement of external-CVCs. Therefore, only the latter 19 patients were considered to have been at risk. The attack rate for external-CVC placements was 31.6% (*n* = 6/19) with a lethality of 0%. No new cases were diagnosed after the IV-team nurse assisted in all CVC insertions, the interventional radiologists received feedback on the incident, and the non-sterile, contaminated solution was removed. The outbreak lasted from July 15 to July 27, 2022. The epidemic curve is shown in Fig. 2.

## Discussion

We report an outbreak of CR-*P. fluorescens* in CRBSI in an IRU of a cancer center, affecting five patients (six events) who underwent external-CVC insertion between June 21 and July 20, 2022. The investigation revealed breaches in the three-decade standing-standardized CVC procedure.

Although *P. fluorescens* has been linked to pseudobacteremias due to contamination of laboratory supplies [5], a total of three confirmed *P. fluorescens* outbreaks have been reported in the last 15 years, all of them involving healthcare associated bloodstream infections. The first one to be described was a multistate outbreak detected in the United States, which was characterized by 80 cases of bacteremia in which the source was found to be a contaminated compounded heparinized saline intravenous flush [6]. The delay between exposure and bacteremia onset was of at least 84 days in 41% of the cases; in contrast, in the outbreak we report, the maximum delay was 24 days from CVC insertion to the diagnosis of CRBSI. This patient was the index case of our outbreak, which can possibly be explained by an exposure to a lower inoculum size due to more recent contamination of the irrigation solution. Benito et al., reported in 2012 an outbreak of six patients in Spain, and Oba et al. in 2017 an outbreak of 13 patients with an attack rate of 40% in Japan, both in coronary care units with isolation of P. fluorescens in contaminated ice baths used for cardiac output determinations [7, 8], as in the outbreak here presented, a breach in the usual procedure was the root to the outbreaks’ genesis. Additionally, Hsueh et al. reported in 1998 the only outbreak described in oncologic patients; however, the source could not be confirmed since the bacteria were not isolated in any of the suspected infusion fluids and disinfectants [9]. Thus, we report the first CRBSI outbreak caused by P. fluorescens in patients with hematologic malignancies.

*P. fluorescens* is common in aquatic environments; however, in instances, as described in this outbreak, it can become an opportunistic pathogen [10]. The resistance pattern of the isolated CR-*P. fluorescens* is of great concern [11]. This outbreak underlines the urgent need to guarantee sterile standardized procedures during CVC insertion to assure the quality of the delivered product, in this case, a healthcare service. Preventing outbreaks is intrinsically linked to the broader issue of preventing and curbing the rise in AMR.

## Conclusions

Rupturing a long-standing standardized CVC placement procedure, resulted in an outbreak of six CR-*P. fluorescens* CRBSI. Standardization of invasive care procedures will allow, like franchises, to obtain the best and most reproducible service, contributing to the prevention of healthcare-associated infections, avoiding antibiotic use, and, thus, antimicrobial pressure. Standardization of healthcare procedures includes highly trained personnel, good-quality supplies, and protocolized procedures.

## Data Availability

No datasets were generated or analysed during the current study.

## Abbreviations

AMR: Antimicrobial resistance
CR: Carbapenem-resistant
CRBSI: Catheter-related bloodstream infection
CVC: Central venous catheter
INCan: Instituto Nacional de Cancerología
IPC: Infection prevention and control
IRU: Interventional radiology unit
IV: Intravenous

## References

[1] Antimicrobial Resistance. World Health Organization. November 21, 2023. Accessed 22 Nov 2023. https://www.who.int/es/news-room/fact-sheets/detail/antimicrobial-resistance.

[2] Volkow P, Sanchez- Mejorada G, de la Vega SL, Vazquez C, Tellez O, Baez RM (1994). Experience of an intravenous therapy team at the Instituto Nacional de Cancerología (Mexico) with a long-lasting, low-cost silastic venous catheter. Clin Infect Dis.

[3] Volkow P, Vázquez C, Téllez O, Aguilar C, Barrera L, Rodríguez E (2003). Polyurethane II catheter as long-indwelling intravenous catheter in patients with cancer. Am J Infect Control.

[4] Volkow P, Tellez O, Vazquez C, Aguilar C, Valencia M, Barrera L (1997). A single, double lumen high-flow catheter for patients undergoing peripheral blood stem cell transplantation. Experience at the National Cancer Institute in Mexico. Bone Marrow Transplant.

[5] Smith J, Ashhurst-Smith C, Norton R (2002). Pseudomonas fluorescens pseudobacteraemia: a cautionary lesson. J Paediatr Child Health.

[6] Gershman MD, Kennedy DJ, Noble-Wang J (2008). Multistate outbreak of Pseudomonas fluorescens bloodstream infection after exposure to contaminated heparinized saline flush prepared by a compounding pharmacy. Clin Infect Dis.

[7] Benito N, Mirelis B, Luz Gálvez M (2012). Outbreak of Pseudomonas fluorescens bloodstream infection in a coronary care unit. J Hosp Infect.

[8] Oba Y, Nakajima T, Ogida C, Kawanami M, Fujiwara M, Matsumura I (2017). Longitudinal nosocomial outbreak of Pseudomonas fluorescens bloodstream infection of 2 years' duration in a coronary care unit. Am J Infect Control.

[9] Hsueh PR, Teng LJ, Pan HJ (1998). Outbreak of Pseudomonas fluorescens bacteremia among oncology patients. J Clin Microbiol.

[10] Silverio MP, Kraychete GB, Rosado AS, Bonelli RR (2022). Pseudomonas fluorescens complex and its intrinsic, adaptive, and acquired antimicrobial resistance mechanisms in pristine and human-impacted sites. Antibiotics (Basel).

[11] Antimicrobial Resistance Collaborators (2022). Global burden of bacterial antimicrobial resistance in 2019: a systematic analysis. Lancet.

